# Integrated Conservation Strategies for the Oravka Chicken: Advances in Cryopreservation Technologies for Endangered Poultry Genetic Resources—A Literature Review

**DOI:** 10.3390/ani16152377

**Published:** 2026-08-03

**Authors:** Andrea Svoradová, Jaromír Vašíček, Lenka Kuželová, Petr Sláma, Ľubomír Ondruška, Peter Chrenek, Francesco Vizzarri

**Affiliations:** 1 Division of Animal Nutrition and Small Livestock, Department of Animal Production, National Agricutural and Food Centre, 951 41 Lužianky, Slovakia; andrea.svoradova@nppc.sk (A.S.); lubomir.ondruska@nppc.sk (Ľ.O.); 2 Division of Animal Genetics and Reproduction, Department of Animal Production, National Agricutural and Food Centre, 951 41 Lužianky, Slovakia; jaromir.vasicek@nppc.sk (J.V.);; 3 Institute of Biotechnology, Faculty of Biotechnology and Food Sciences, Slovak University of Agriculture, 949 76 Nitra, Slovakia; 4 AgroBioTech Research Centre, Slovak University of Agriculture in Nitra, 949 76 Nitra, Slovakia; lenka.kuzelova@uniag.sk; 5 Laboratory of Animal Immunology and Biotechnology, Department of Animal Morphology, Physiology and Genetics, Faculty of AgriSciences, Mendel University in Brno, 613 00 Brno, Czech Republic; petr.slama@mendelu.cz

**Keywords:** Oravka chicken, animal genetic resources, stem cells, semen, cryopreservation, gene bank

## Abstract

Local poultry breeds represent an essential component of agricultural biodiversity and play an important role in ensuring the resilience of livestock production systems under changing environmental conditions. The Slovak national chicken breed Oravka is a dual-purpose genotype adapted to mountain and sub-mountain production environments and classified as an endangered genetic resource. The conservation of this breed therefore requires integration of traditional breeding programmes with modern reproductive biotechnologies. The present literature review summarises the origin, breeding characteristics and adaptive potential of the Oravka breed and provides a comprehensive overview of current ex situ conservation strategies based on cryopreservation of reproductive and stem cells. Special attention is devoted to the cryopreservation of primordial germ cells, mesenchymal stem cells and rooster spermatozoa, which represent key biological material stored within national gene bank infrastructure. Differences in cryosurvival between blastodermal cells and germline stem cells are discussed together with methodological limitations of avian embryo cell preservation. The review highlights the importance of combining in situ and ex situ conservation approaches and emphasises the role of poultry gene banks as strategic tools for safeguarding genetic diversity under climate change conditions. The Oravka breed represents a valuable model for integrated conservation strategies linking biodiversity protection, reproductive biotechnology and sustainable poultry production in Europe.

## Simple Summary

Local chicken breeds are an important part of agricultural biodiversity because they carry unique genetic traits that help animals adapt to changing environments, resist diseases, and thrive under traditional farming conditions. However, many of these breeds are becoming endangered as they are replaced by highly productive commercial chickens. This review focuses on the Slovak national chicken breed Oravka and explains how traditional breeding programmes can be combined with modern methods for preserving genetic material. It summarises current knowledge on storing reproductive cells and other valuable cells at very low temperatures for future use, highlighting the advantages and limitations of different approaches. Recent advances show that preserving cells capable of producing the next generation offers new opportunities for safeguarding the complete genetic heritage of endangered breeds, while storing semen and other cell types provides additional security for long-term conservation. Combining these methods with the maintenance of live breeding populations offers the best strategy for protecting valuable genetic resources. Conserving native breeds such as Oravka helps preserve biodiversity, supports sustainable agriculture, and provides genetic resources that may become increasingly important for food production under future environmental and climate challenges. This approach is also aligned with the priorities of the European Union in the areas of the conservation of genetic resources, biodiversity, and sustainable food systems, as outlined in the European Green Deal and the EU Biodiversity Strategy for 2030.

## 1. Introduction

The conservation of biodiversity of animal genetic resources is currently one of the key priorities in sustainable agriculture and modern biotechnologies. Various environmental and socio-economic factors, including climate change, intensification of livestock production systems, and loss of natural habitats, threaten the genetic diversity of animal species and consequently the stability of global food security. Therefore, maintaining animal genetic resources is essential not only from the perspective of protecting natural heritage but also as a strategic tool for future research and innovative applications in biotechnology and breeding programmes [1]. Currently, two main conservation strategies for animal genetic resources are recognised: in situ conservation (preservation within the original production environment) and ex situ conservation (preservation outside the original environment). Modern biotechnological approaches increasingly focus on ex situ conservation through cryopreservation, which enables long-term storage of biological material at ultra-low temperatures. Cryopreservation represents an indispensable tool for safeguarding endangered breeds and genetic lines and involves a wide spectrum of biological materials ranging from embryos, spermatozoa and oocytes to stem cells and tissues [2].

This literature review focuses on the cryopreservation of embryonic and adult stem cells and spermatozoa of the Oravka chicken breed, a Slovak national poultry breed representing an important component of the cultural and genetic heritage of the country. Although the review summarises advances in avian cryobiology in general, the Oravka breed is used as a representative case study because recent experimental work has generated one of the most comprehensive collections of cryopreserved biological resources for an endangered European chicken breed. Therefore, the review combines general methodological advances with their practical application to the conservation of a national genetic resource. Poultry plays an important role worldwide not only in food production but also as a model organism in scientific research. In the context of climate change, research aimed at mitigating the negative effects of heat stress on poultry health and reproductive performance is becoming increasingly relevant. Despite significant progress in cryopreservation techniques, optimisation of preservation methodologies remains a major challenge. It is therefore necessary to investigate factors influencing the success of cryopreservation procedures, including the selection of appropriate cryoprotectants, optimisation of freezing and thawing rates, and evaluation of post-thaw cell viability. Assessment of cell quality after thawing represents a critical step for ensuring their functional integrity and biological relevance, while modern biotechnological approaches provide new analytical tools supporting detailed evaluation and innovation in this field [3]. The aim of this review is not to summarise cryopreservation technologies in isolation but to evaluate how currently available in situ and ex situ conservation strategies can be integrated to support long-term preservation of endangered poultry breeds, using the Slovak Oravka chicken as a representative case study.

## 2. Materials and Methods

To provide a comprehensive overview of the Oravka chicken as one of Slovakia’s national animal genetic resources, a systematic literature review was performed. The review focused on summarising current knowledge of the breed, with particular emphasis on conservation strategies, ex situ preservation, and modern cryopreservation approaches applicable to safeguarding its genetic diversity. Relevant publications were retrieved from the ScienceDirect, PubMed, Scopus, Google Scholar, and Web of Science databases. The literature search was conducted using combinations of the following keywords: “Oravka chicken”, “animal genetic resources”, “stem cells”, “semen”, “cryopreservation”, and “gene bank”. The initial search identified approximately 160 publications. The retrieved studies were screened according to predefined inclusion criteria, including relevance to the animal model, biological materials used, research topic, main contribution to the research area, and the primary outcomes investigated. Publications that did not meet these criteria or were not directly relevant to the scope of this review were excluded. Following the selection process, 88 studies were included in the final analysis. The selected literature provided a comprehensive basis for evaluating the current status of the Oravka chicken breed and highlighted recent advances in conservation biology, reproductive biotechnology, and cryobanking. The findings presented in this review are intended to serve as a valuable resource for researchers, students, breeders, and professionals involved in the conservation and sustainable utilisation of animal genetic resources.

## 3. Conservation Strategies

### 3.1. Native Chicken Breeds as Strategic Genetic Resources Under Climate Change

Over the past 250 years, substantial changes have occurred in the diversity of farm animal populations and in the development of specialised livestock breeds, accompanied by systematic recording of pedigrees and production traits. These developments contributed to fixation of breed-specific characteristics and improvement of productivity. However, already at the end of the 19th century, several traditional breeds began to be replaced by more productive populations [4].

During the 19th century, the expansion of railway and steamship transport enabled long-distance movement of livestock. After World War II, the introduction of artificial insemination technologies in cattle and pig breeding further accelerated the global spread of a limited number of highly productive commercial breeds such as Holstein cattle and Large White pigs, which currently dominate worldwide livestock production systems [5].

Understanding the historical development and distribution of livestock populations is essential for their effective utilisation and long-term preservation as animal genetic resources. Diversity of animal genetic resources plays a key role in ensuring successful livestock production under diverse environmental conditions and supports the availability of a wide range of animal products. It also provides flexibility in adapting breeding objectives in response to changing market conditions and emerging global challenges.

Local breeds often have significant cultural importance for the communities in which they were developed. Breeds shaped primarily by natural selection are typically well adapted to their environments and play an essential role in ecological systems by controlling vegetation growth and supporting biodiversity conservation. For example, the Engadin sheep breed, which nearly disappeared during the 1980s, is currently used to maintain historical alpine pastures by grazing invasive shrubs.

Grazing livestock also contribute to carbon sequestration by removing plant biomass and promoting regrowth, thereby enhancing the transfer of atmospheric carbon into soil organic matter. Increased livestock diversity improves preparedness for future challenges such as climate change. Access to diverse genetic traits may enhance adaptation capacity to adverse climatic conditions and emerging diseases. Animals possessing specific adaptive characteristics such as disease resistance, tolerance to parasites, ability to utilise low-quality feed resources or capacity to survive under dry or hot climatic conditions may significantly contribute to improved resilience of agricultural production systems under changing environmental conditions.

In situations where certain breeds have limited opportunities for sustainable utilisation or are kept under unfavourable production conditions, targeted conservation programmes are required to preserve their genetic diversity. Several approaches are currently applied, including in situ conservation through maintenance of live populations and ex situ conservation using cryopreservation of genetic material [6]. In many cases, both strategies are combined. Continued research on conservation methodologies and technologies remains essential, particularly for less common livestock species, and requires sustained financial investment. Many countries currently operate national programmes for conservation of animal genetic resources at least for selected livestock species and breeds [1].

The term animal genetic resources (AnGR) is commonly used to describe animal species that are used, or may potentially be used, for food and agricultural production [7]. One of the main reasons for increasing international interest in the status and management of animal genetic resources is the need to ensure that livestock populations continue to fulfil their essential roles in supporting livelihoods and food production worldwide while preserving the genetic diversity embedded in livestock biodiversity [1].

The Global Plan of Action for Animal Genetic Resources was adopted in Interlaken, Switzerland, in 2007 by 109 countries as the first internationally agreed framework for the management of animal genetic resources and was subsequently endorsed by all FAO member states (191 countries and the European Community). The plan emphasises that understanding diversity, distribution, basic characteristics and the status of national animal genetic resources is essential for their effective and sustainable utilisation, development and conservation.

More than 90% of global livestock production relies on a limited number of species including pigs, poultry, cattle, sheep, goats and buffalo. Other widely distributed domesticated species include rabbits, turkeys, ducks, geese, guinea fowl, quails, pigeons and ostriches.

For effective management of animal genetic resources in Europe, two important databases have been developed by the European Federation of Animal Science (EAAP) and the European Farm Animal Biodiversity Information System (EFABIS). EFABIS serves as a platform for exchange of national data on animal genetic resources provided by national coordinators across Europe and contributes to the Domestic Animal Diversity Information System (DAD-IS) managed by FAO. Its objective is to support the development of a sustainable management structure for European networks operating at national, European and international levels [8].

Conservation strategies for animal genetic resources can be implemented either as in situ or ex situ conservation, with ex situ approaches further divided into ex situ in vivo and ex situ in vitro conservation [5].

- In situ conservation involves maintenance of live animal populations within production systems in a way that ensures optimal utilisation of genetic diversity in the short term while preserving long-term sustainability of the population [9,10].
- Ex situ conservation involves preservation of genetic resources outside their original production environment and includes maintenance of live animals (ex situ in vivo) as well as cryopreservation of biological material (ex situ in vitro).
- Ex situ in vivo conservation refers to maintenance of live animals outside their natural or traditional production environment. However, like in situ conservation, genetic drift may occur within isolated populations, which may not guarantee preservation of the full genetic variability of the breed. Therefore, conservation of live populations should be complemented by cryopreservation of genetic material [10].
- Ex situ in vitro conservation includes deep freezing of spermatozoa, oocytes, embryos or tissues for potential future use in breeding or regeneration programmes [11].
- An additional conservation concept referred to as in libro describes preservation of knowledge about already extinct breeds or their specific characteristics within historical and scientific documentation [12].

Although in situ conservation is often considered the most appropriate approach because it maintains breeds in a dynamic evolutionary state, commercially important breeds are frequently exposed to intensive selection pressure, while less economically important breeds are often maintained in small populations vulnerable to genetic drift or extinction. Therefore, integration of in situ and ex situ conservation strategies represents the most effective approach for long-term preservation of animal genetic resources [13].

### 3.2. Stem Cells

Stem cells are undifferentiated cells capable of differentiating into various cell types of the organism and possessing the ability of self-renewal. According to their origin, stem cells are classified as embryonic or adult. Their developmental potential decreases progressively, meaning that a unipotent stem cell cannot differentiate into as many cell types as a pluripotent stem cell. Totipotent stem cells can divide and differentiate into all cell types of the organism. Totipotency represents the highest differentiation potential and enables cells to generate both embryonic and extraembryonic structures. One example of a totipotent cell is the zygote, which is formed after fertilisation of the oocyte by the spermatozoon. These cells can later develop into any of the three germ layers or contribute to placental formation. After approximately four days, the inner cell mass of the blastocyst becomes pluripotent and serves as a source of pluripotent cells. Pluripotent stem cells (PSCs) can generate cells of all three germ layers but not extraembryonic structures such as the placenta. These include embryonic stem cells (ESCs), which are present in the inner cell mass of the embryo. Following fusion of the sperm and egg, the blastocyst is formed. Its inner wall is characterised by the presence of stem cells, specifically embryonic stem cells. Blastocysts consist of two distinct cell types: the inner cell mass (ICM), which develops into the epiblast and initiates fetal development, and the trophectoderm (TE). The TE continues development and forms extraembryonic supporting structures necessary for successful embryogenesis, such as the placenta. While the TE begins forming specialised support tissues, the ICM cells remain undifferentiated, fully pluripotent, and proliferative [14]. The pluripotency of stem cells enables them to give rise to any cell type in the organism. Embryonic stem cells originate from the ICM. During embryogenesis, cells form the three germ layers, endoderm, mesoderm, and ectoderm, each of which gives rise to differentiated cells and tissues of the fetus and, later, the adult organism [15]. Once ESCs differentiate into one of the germ layers, they become multipotent stem cells whose functionality is restricted to cell types derived from that germ layer. This stage is relatively short during organismal development. Later, pluripotent stem cells persist throughout the organism as undifferentiated cells, and their key properties are proliferation through generation of additional stem cells and differentiation into specialised cells under specific physiological conditions. Induced pluripotent stem cells (iPSCs) represent another example of pluripotent cells. iPSCs are artificially generated from somatic cells and possess properties like those of PSCs. Their cultivation and application are considered highly promising for present and future regenerative medicine.

Multipotent stem cells have a narrower differentiation spectrum than PSCs. One example is the hematopoietic stem cell (HSC), which can give rise to several types of blood cells. After differentiation, the HSC becomes an oligopotent cell, and its differentiation potential is further restricted to cells within its lineage. Oligopotent stem cells can differentiate into only a few cell types. For example, a myeloid stem cell can differentiate into white blood cells but no longer into red blood cells. Unipotent stem cells have the narrowest differentiation capacity but retain the ability to undergo repeated division. This property makes them suitable candidates for therapeutic use in regenerative medicine. Such cells can produce only one cell type, such as dermatocytes [15].

## 4. Embryonic Stem Cells

Successful ex situ conservation of poultry genetic resources relies on the preservation of biological materials capable of transmitting or representing the genetic diversity of a breed. Among these, embryonic stem cells, primordial germ cells, mesenchymal stem cells and spermatozoa differ in their biological properties, conservation potential and practical applications. The following sections summarise their characteristics and relevance for integrated poultry gene banks.

### 4.1. Blastodermal Cells

Over recent decades, intensive research has increasingly focused on conservation of genetic diversity in endangered domestic animal breeds, as the progressive decline in genetic variability and the growing dominance of commercial hybrid poultry lines represent major threats to long-term sustainability of animal genetic resources worldwide [16]. Preservation of genetic material in gene banks therefore represents one of the key tools supporting long-term conservation of livestock biodiversity.

In poultry species, ex situ in vivo conservation strategies remain the most widely used approach. However, continuous industrialisation of poultry production systems and strong selection pressure for productivity traits contribute to reductions in effective population sizes of local breeds. Although cryopreservation of spermatozoa represents an important conservation tool, it enables preservation of only the paternal genome and therefore cannot ensure complete restoration of genetic diversity. For this reason, increasing attention has been directed towards embryonic and stem cell populations capable of transmitting genetic information between generations. Among these cellular resources, blastodermal cells (BCs), embryonic stem cells (ESCs) and primordial germ cells (PGCs) represent promising candidates for whole-genome preservation strategies in poultry gene bank programmes. However, their suitability for long-term cryoconservation differs substantially depending on their biological characteristics and cryoresistance.

BCs represent a pluripotent embryonic cell population located in the germinal disc of the avian blastoderm and are therefore considered a potential source of cells capable of contributing to both somatic and germline lineages. Early embryonic development in poultry is commonly described using the Eyal-Giladi and Kochav (EGK) staging system, which characterises intrauterine development prior to oviposition [17]. At stage EGK X, the blastoderm contains approximately 50,000–60,000 undifferentiated progenitor cells with the capacity to differentiate into multiple cell lineages. Because of their pluripotent character and accessibility at early developmental stages, BCs have been considered a potential tool for preservation of poultry genetic resources. Under appropriate in vitro culture conditions supported by feeder layers and growth factors such as fibroblast growth factor and leukaemia inhibitory factor, BCs can maintain an undifferentiated state and contribute to both somatic and germline chimeras following transfer into recipient embryos. Despite their theoretical potential for genome preservation, practical application of BCs in poultry gene bank programmes remains limited. The efficiency of chimera production following transplantation of cultured BCs into recipient embryos is generally low, mainly due to reduced migratory capacity towards developing gonads after in vitro culture [18]. In addition to these biological limitations, cryopreservation efficiency of BCs appears to be strongly influenced by their intracellular composition. Earlier studies evaluating BC survival after freezing frequently relied on trypan blue staining and reported relatively high post-thaw viability [19,20,21]. However, these estimates may be affected by the presence of lipid droplets within the germinal disc structure, which are impermeable to trypan blue and may therefore lead to overestimation of cell survival.

More recent fluorescence-based approaches combining nuclear markers such as DRAQ5 with viability dyes and flow cytometric analysis have demonstrated substantially lower post-thaw survival rates of BCs and revealed extensive ultrastructural damage associated with their lipid-rich cytoplasmic composition [22]. These findings suggest that intracellular lipid content represents a major obstacle limiting cryoprotectant penetration and contributes to membrane disruption during freezing. Importantly, alternative freezing strategies, including controlled slow freezing and vitrification, have not significantly improved post-thaw survival of BCs under current experimental conditions. These observations indicate that BCs may have limited suitability for long-term storage in poultry gene bank systems using currently available cryopreservation technologies. Another important limitation affecting interpretation of BC viability concerns the use of Annexin V staining for detection of apoptotic cells. Because Annexin V binds phospholipid components of cellular membranes, nonspecific interactions with lipid droplets derived from yolk material may occur. Combined staining with nuclear markers such as DRAQ5 or DAPI therefore represents a more reliable approach for distinguishing true apoptotic BC populations from non-cellular lipid structures [23].

Taken together, although BCs represent an attractive pluripotent cell population from a theoretical perspective, their practical application for long-term genome preservation in poultry gene bank systems remains limited. These findings highlight the importance of identifying alternative germline-competent cell populations with higher cryoresistance and improved suitability for restoration of endangered poultry breeds such as the Oravka chicken within integrated national gene bank conservation strategies.

### 4.2. Primordial Germ Cells

While BCs have long been considered promising candidates for genome preservation because of their pluripotency, current evidence indicates that their practical application in long-term poultry gene banking remains constrained by biological and technical limitations due to their relatively low post-thaw survival and reduced germline transmission efficiency. These limitations highlight the importance of identifying alternative germline-competent cell populations with higher cryoresistance and improved suitability for restoration of endangered poultry breeds.

In this context, primordial germ cells (PGCs) represent one of the most promising cellular resources currently available for genome preservation in poultry species [20]. Unlike BCs, PGCs are direct precursors of gametes and therefore retain the unique ability to transmit genetic information to subsequent generations through germline chimerism [24]. Because of these characteristics, PGCs are increasingly recognised as key biological material for long-term conservation of endangered poultry breeds in gene bank systems. Optimisation of isolation, characterisation and cryopreservation protocols for PGCs therefore represents an essential step in the development of effective ex situ preservation strategies.

Previous studies have demonstrated that gonadal PGCs isolated from embryos at developmental stages around HH 28–29 can be successfully cryopreserved using standard slow-freezing protocols with dimethyl sulfoxide as a cryoprotective agent, followed by long-term storage in liquid nitrogen [25]. Because embryonic gonadal tissue represents a heterogeneous cellular population, reliable evaluation of post-thaw viability is critical for assessing the suitability of PGCs for gene bank applications. Conventional trypan blue staining has been widely used for this purpose; however, fluorescence-based approaches such as propidium iodide staining provide more accurate discrimination between viable and damaged cells. Differences between these methods have been attributed mainly to variations in membrane permeability and dye exposure time, which may lead to overestimation of cell mortality when trypan blue staining is applied [26,27]. These observations support the increasing use of fluorescence microscopy as a more reliable tool for evaluation of PGC survival after cryopreservation.

Importantly, comparative cryopreservation studies have demonstrated substantially higher survival rates of PGCs than BCs, with post-thaw viability reaching approximately 70%, further supporting their suitability as a robust cellular resource for poultry gene bank applications [28]. Ultrastructural analyses performed using transmission electron microscopy confirmed preservation of nuclear and cytoplasmic integrity following freezing, indicating good tolerance of PGCs to standard cryopreservation procedures. In addition to viability assessment, confirmation of the stem cell phenotype represents an important step in characterisation of isolated PGC populations. Aldehyde dehydrogenase (ALDH) activity has been widely recognised as a functional marker of stemness associated with regulation of proliferation, differentiation and protection against oxidative stress [29,30,31]. High intracellular ALDH activity (approximately 85%) has been detected in cryopreserved poultry PGC populations [28], representing the first evidence of strong ALDH expression in avian germline precursor cells. Comparable ALDH activity levels have previously been described in haematopoietic stem cells and other progenitor cell populations in mammals [32,33,34], supporting the interpretation that ALDH represents a conserved marker of stem cell functionality across species.

Recent work on the Slovak Oravka breed has further expanded the application of PGC technology from cryopreservation towards comprehensive gene bank establishment [35]. Stable PGC lines were successfully derived from circulating embryonic PGCs collected from individual Oravka embryos, representing both sexes, and subsequently cryopreserved for long-term storage. The established cell lines retained normal morphology, stable proliferation during extended in vitro culture, expression of germ cell- and pluripotency-associated markers (DDX4, DAZL and POU5F3), normal karyotypes after prolonged cultivation, and typical ultrastructural characteristics observed by transmission electron microscopy. Functional validation demonstrated that cryopreserved and subsequently cultured PGCs preserved their migratory ability and efficiently colonised the gonads of recipient embryos following transplantation, confirming maintenance of their biological competence after freezing. The establishment of both male and female PGC lines also enables preservation of the complete diploid genome, representing a major advantage over sperm cryobanking, which conserves only the paternal genome [35].

Taken together, these findings confirm that PGCs represent a highly suitable cellular resource for preservation of poultry genetic material and provide a practical and biologically efficient alternative to BCs for long-term storage of endangered breeds such as the Oravka chicken within national gene bank infrastructures. Recent advances in establishing, validating and cryobanking breed-specific PGC lines further demonstrate that this technology has progressed from experimental proof-of-concept to a practical conservation tool that can support future regeneration of endangered poultry populations through germline chimera technologies [35].

### 4.3. Bone Marrow-Derived Mesenchymal Stem Cells

Bone marrow represents an important reservoir of adult stem and progenitor cell populations and provides a specialised microenvironment supporting their maintenance, proliferation and differentiation. Among these populations, mesenchymal stem cells (MSCs) play a central role in skeletal development and tissue regeneration through their ability to differentiate into osteoblasts, chondrocytes, adipocytes and several additional mesoderm-derived cell types [36]. In poultry species, bone marrow-derived MSCs therefore represent a valuable source of undifferentiated progenitor cells with potential applications not only in regenerative biology but also in preservation of animal genetic resources. Compared with embryonic stem cells, adult stem cells offer several practical advantages for conservation-oriented biotechnology, particularly with respect to accessibility, ethical acceptability and long-term applicability. Because they can be isolated from postnatal tissues without the need for embryo manipulation, adult stem cells represent a feasible and sustainable source of genetic material for cryobanking strategies supporting preservation of endangered breeds [6]. In vivo, their biological activity is regulated by signals originating from the stem cell niche, which controls their proliferation, survival and differentiation potential and enables their participation in tissue homeostasis and regeneration [37,38,39]. Among adult stem cell populations, MSCs are of particular interest because of their plastic adherence, strong proliferative capacity, multilineage differentiation ability and secretion of cytokines and growth factors involved in tissue repair and hematopoietic regulation [40,41,42]. MSCs have been identified in a wide range of tissues, including bone marrow, adipose tissue, cartilage, skeletal muscle, dental tissues, endometrium, liver and several perinatal tissues [43,44,45]. In poultry research, MSCs are increasingly recognised as valuable experimental models for studies on skeletal development, locomotor disorders and regenerative processes, particularly in intensive broiler production systems where rapid growth has been associated with musculoskeletal abnormalities [46,47]. Because MSCs represent progenitors of bone, cartilage and muscle tissues, they are also highly relevant for the development of supportive regenerative and biobanking strategies [48].

Recent studies have further expanded the potential applications of poultry MSCs, including their use in three-dimensional culture systems, feeder-layer technologies, in vitro meat production, disease modelling and cryopreservation-based conservation approaches. Their capacity to migrate to damaged tissues, proliferate efficiently and secrete bioactive molecules and extracellular vesicles further increases their value as biologically active cell populations supporting tissue homeostasis and repair [49]. These properties make MSCs attractive not only as experimental tools but also as complementary cellular resources for long-term preservation of valuable poultry genetic material. Bone-derived MSC populations have been successfully isolated and cryopreserved in both commercial poultry hybrids and native breeds such as the Oravka chicken [50] using standard slow-freezing protocols based on dimethyl sulfoxide as a cryoprotective agent, followed by long-term storage in liquid nitrogen [51,52]. During in vitro culture, poultry MSCs typically exhibit characteristic morphological changes from oval-shaped cells to spindle-shaped fibroblast-like colonies, accompanied by rapid proliferation and achievement of high confluence within several days, which is consistent with previously reported observations in chicken MSC cultures [53,54,55,56,57,58,59,60].

High post-thaw viability of poultry MSCs has been demonstrated using multiparametric flow cytometric approaches such as Annexin V/7-AAD staining, confirming preservation of cell integrity after freezing. Phenotypic characterisation of these cells revealed strong expression of canonical MSC-associated surface markers, including CD73, CD90, CD105, CD29, CD44 and CD146, together with absence of hematopoietic markers CD34 and CD45, supporting their mesenchymal identity [53,54,55]. In addition, expression of intracellular cytoskeletal markers such as vimentin, desmin and actin has been confirmed using flow cytometry and confocal microscopy, thereby expanding the available phenotypic profile of poultry bone-derived MSC populations [50]. Further support for stem cell functionality has been provided by detection of high aldehyde dehydrogenase (ALDH) activity, which represents a recognised marker of undifferentiated stem cell populations involved in regulation of proliferation, differentiation and protection against oxidative stress [61]. Multilineage differentiation capacity into osteogenic, adipogenic and chondrogenic lineages has also been consistently demonstrated in poultry MSC cultures, confirming preservation of their functional properties following cryopreservation [50,53,54,55].

Ultrastructural analyses further indicate that cryopreserved MSCs retain general cellular integrity, including preservation of nuclear morphology and intracellular organelles after thawing, supporting their suitability for long-term storage in poultry gene bank systems. Although MSCs do not provide direct germline transmission potential comparable to PGCs, they represent an important complementary component of integrated cryoconservation strategies by preserving somatic genomic information and providing biologically versatile material for future research, biotechnology and conservation-related applications. In native breeds such as the Oravka chicken, successful cryopreservation of bone-derived MSCs therefore represents an important extension of multi-level cellular conservation strategies supporting long-term safeguarding of endangered poultry genetic resources (Table 1).

## 5. Rooster Spermatozoa as a Core Resource for Poultry Gene Bank Conservation

Cryopreservation of poultry spermatozoa represents one of the most practical and widely applicable approaches for preservation of avian reproductive genetic material. In contrast to mammalian species, where both oocytes and embryos are routinely used for ex situ conservation programmes, preservation of female germ cells in birds remains technically limited due to the large yolk-rich structure of the avian oocyte [62]. As a consequence, sperm cryopreservation has become a cornerstone of poultry gene banking and an essential strategy for long-term conservation of endangered breeds. Cryopreserved semen enables storage of viable male gametes outside the organism and their later use in artificial insemination or breed restoration programmes. However, despite its practical importance, freezing procedures are associated with substantial cryoinjury affecting post-thaw motility, membrane integrity and fertilising capacity [63,64].

The efficiency of rooster sperm cryopreservation is influenced by multiple interrelated factors, including extender composition, type and concentration of cryoprotective agents (CPAs), osmolarity, pH, intracellular water content, membrane lipid composition and thawing conditions [65]. Among these variables, the choice of CPA plays a particularly important role because it directly affects the extent of osmotic and physicochemical stress during freezing. Permeating CPAs such as dimethyl sulfoxide, glycerol, ethylene glycol, propylene glycol and methanol reduce intracellular ice formation by penetrating the sperm cell, whereas non-permeating cryoprotectants such as polyvinylpyrrolidone, hydroxyethyl starch or sugars primarily stabilise the extracellular environment. Nevertheless, all CPAs exhibit some degree of cytotoxicity, and excessive exposure may result in membrane destabilisation, reduced motility or acrosomal alterations, which represent major manifestations of cryodamage [66].

These limitations are particularly relevant in poultry species because rooster spermatozoa exhibit high sensitivity to cold shock and oxidative stress [67,68]. Their elongated morphology reduces cytoplasmic volume, and membrane composition enriched with phospholipids and polyunsaturated fatty acids increases susceptibility to osmotic imbalance and lipid peroxidation during freezing. In addition, breed-specific differences in sperm cryotolerance have been reported, indicating that optimisation of preservation protocols should be adapted to native genotypes rather than directly extrapolated from commercial hybrid lines [69,70].

Because of these biological constraints, robust post-thaw evaluation of sperm quality represents an essential component of poultry semen cryobiology. Conventional microscopic assessment of motility provides only limited information and may fail to detect sublethal cellular damage affecting fertilising capacity after thawing. For this reason, modern analytical approaches such as computer-assisted sperm analysis (CASA) and flow cytometry have become key tools for objective evaluation of post-thaw sperm quality.

CASA enables quantitative assessment of total and progressive motility together with detailed kinematic parameters including curvilinear velocity (VCL), straight-line velocity (VSL), average path velocity (VAP), linearity (LIN), straightness (STR), wobble (WOB), amplitude of lateral head displacement (ALH) and beat-cross frequency (BCF). These parameters provide detailed information on sperm movement characteristics and allow comparison between fresh and cryopreserved samples across breeds and freezing protocols. However, motility assessment alone does not fully reflect membrane integrity or fertilising potential. Flow cytometry therefore represents an important complementary approach for multiparametric evaluation of sperm physiology at the single-cell level. In poultry semen, flow cytometric analyses commonly include assessment of plasma membrane integrity, apoptosis-like membrane alterations, acrosome status, mitochondrial membrane potential, reactive oxygen species production, capacitation-related changes and DNA damage. Viability is typically evaluated using SYBR-14 in combination with propidium iodide or 7-AAD, whereas apoptosis-like changes may be detected using Annexin V or YO-PRO-1. Acrosome integrity can be assessed using lectins such as PNA or PSA, mitochondrial function using dyes such as rhodamine 123 or JC-1 and oxidative stress using probes including H_2_DCFDA, DHE, MitoSOX or C11-BODIPY 581/591 [61,71,72,73,74]. Because oxidative stress represents one of the major drivers of post-thaw sperm dysfunction, these analytical approaches are particularly relevant for evaluation of avian semen cryosurvival.

Studies performed on native breeds such as the Oravka chicken further confirm that optimisation of cryoprotectant selection represents a critical determinant of post-thaw sperm quality. Comparative analyses using dimethyl sulfoxide, ethylene glycol and glycerol have demonstrated significant differences in cryosurvival between cryoprotectants, with glycerol providing the highest preservation of total motility after thawing, reaching approximately 45%, which is consistent with previously reported results in poultry species [64,69,75,76,77]. Nevertheless, increased proportions of dead and apoptosis-like sperm populations were observed following freezing regardless of the CPA used, indicating that membrane destabilisation remains a major limiting factor affecting cryosurvival [77].

Multiparametric flow cytometric approaches combining YO-PRO-1, propidium iodide and lectin-based acrosome staining have further demonstrated that freezing procedures induce substantial membrane stress and partial loss of functional integrity in rooster spermatozoa. Introduction of nuclear markers such as DRAQ5 has been shown to improve discrimination between nucleated spermatozoa and non-cellular debris, thereby increasing the accuracy of viability assessment in semen samples containing high levels of seminal plasma contaminants [77].

Ultrastructural analyses performed using transmission electron microscopy indicate that the plasma membrane represents the primary site of cryoinjury during freezing and thawing procedures, which is consistent with previous observations in avian sperm cryobiology [62]. Additional structural factors contributing to cryosensitivity include the elongated morphology of the rooster sperm head, reduced cytoplasmic volume and the high proportion of polyunsaturated fatty acids in sperm membranes, all of which increase susceptibility to lipid phase transitions and oxidative damage during freezing [78,79].

Taken together, these findings confirm that rooster spermatozoa represent a highly relevant and operationally feasible resource for ex situ conservation of poultry genetic material, including endangered native breeds such as Oravka. Although sperm cryopreservation alone cannot ensure preservation of the complete diploid genome in the same way as germline stem cells, it remains the most mature and directly applicable reproductive biotechnology currently available for poultry gene bank systems. Within integrated conservation strategies, rooster sperm therefore represents a core component of avian cryobanking, complementing PGCs and MSCs within multi-level preservation frameworks designed for long-term safeguarding of poultry genetic diversity.

## 6. Conservation of Poultry Genetic Resources: The Oravka Chicken as a Model for Integrated Preservation

### Oravka Chicken as a National Poultry Genetic Resource

The Oravka chicken breed represents one of the most important autochthonous poultry genetic resources of Slovakia. In 1990, the yellow-brown variety was officially recognised as a Slovak national breed, followed by recognition of the white colour variety in 2008. Due to declining population size and increasing pressure from commercial hybrid poultry production systems, the breed has subsequently been classified among endangered genetic resources requiring targeted conservation measures.

The breeding programme of the Oravka breed was originally designed to develop a genotype adapted to the harsh environmental conditions of mountainous and sub-mountain regions of Slovakia, with the ability to efficiently utilise outdoor production systems and pasture-based feeding conditions typical for these areas [13]. The breed was established through a structured crossbreeding programme involving three foundation breeds, Rhode Island Red, White Wyandotte and New Hampshire. Oravka belongs to medium-heavy dual-purpose chicken breeds characterised by balanced egg and meat production performance. Adult body weight ranges between 2.8 and 3.3 kg in roosters and between 2.2 and 2.7 kg in hens [80].

Local poultry breeds such as Oravka represent valuable reservoirs of unique adaptive traits associated with resilience to marginal production environments, disease resistance, and suitability for alternative or extensive production systems. Preservation of such locally adapted genotypes is therefore essential not only for maintaining agricultural biodiversity but also for ensuring long-term sustainability of livestock production under changing environmental and climatic conditions. For endangered breeds with small effective population sizes, conservation strategies must integrate traditional in situ breeding programmes with advanced ex situ reproductive biotechnology approaches.

In addition to in situ conservation through structured breeding programmes, the Oravka breed is included in the ex situ conservation activities of the National Agricultural and Food Centre (NPPC, Luzianky, Slovakia), where representative live populations are maintained within the national animal collection as a complementary safeguard of this endangered genetic resource. This ex situ in vivo approach provides an additional level of security against the loss of genetic diversity while supporting research, education, and future breeding initiatives. Furthermore, as a member of the European Gene Bank Network for Animal Genetic Resources (EUGENA), NPPC actively contributes to the implementation of European best practices for the conservation and sustainable use of livestock genetic resources. The integration of national conservation activities with European collaborative initiatives strengthens the long-term preservation of the Oravka breed and facilitates harmonisation of conservation strategies across Europe.

One of the most promising tools for safeguarding endangered animal genetic resources is the cryopreservation of reproductive and stem cells, which enables long-term storage of genetic material in animal gene banks and supports future restoration of endangered populations [81]. In poultry species, preservation of genetic material is particularly important because of biological limitations associated with cryopreservation of avian oocytes and embryos. Unlike mammalian species, where embryos and oocytes represent standard biological material routinely used for gene bank storage and restoration of genetic variability through assisted reproductive technologies, these approaches are not directly applicable in birds [82].

One of the major constraints in avian genome preservation is the structural complexity of the avian oocyte. Its large size and high lipid and yolk content prevent adequate penetration of cryoprotective agents and promote intracellular ice crystal formation during freezing. Similarly, cryopreservation of avian embryos remains technically challenging due to their meroblastic cleavage pattern and the presence of a large yolk mass acting as a physical barrier to controlled cooling and uniform dehydration during freezing procedures. As a consequence, routine embryo-based genome preservation strategies commonly used in mammals cannot currently be applied in poultry species.

Because these biological constraints limit the use of embryos and oocytes for genome preservation, alternative cellular strategies must be considered for effective ex situ conservation of avian genetic resources. In this context, preservation of reproductive and stem cell populations capable of transmitting genetic information between generations represents a key approach for long-term safeguarding of endangered poultry breeds [83]. Among the most promising cell types currently used in avian gene bank programmes are BCs, PGCs, MSCs and rooster spermatozoa [84].

These cellular resources differ in their biological characteristics, genome preservation capacity and potential applications in breed restoration programmes and together form the basis of integrated multi-level cryoconservation strategies in poultry genetic resource management.

Cryopreservation represents one of the most effective tools currently available for long-term preservation of such cellular resources because it enables secure storage of reproductive and stem cell populations while maintaining their biological functionality for future breeding and research applications [85]. In critically endangered local breeds such as Oravka, preservation of multiple complementary cell types within national gene banks is particularly important, as storage of a single biological material type cannot ensure sufficient long-term genome security. Instead, integrated cryobanking strategies combining germline and somatic cell preservation significantly increase the probability of successful restoration of endangered populations.

In addition to its conservation value, cryopreservation reduces the need for continuous in vitro maintenance of cell cultures, minimises risks associated with contamination and phenotypic instability, and facilitates safe transport of biological material between research institutions and gene bank infrastructures [38,86].

In this context, systematic cryopreservation of multiple cellular resources including BCs, PGCs, MSCs and rooster spermatozoa represents a strategic approach for safeguarding the genetic integrity of the Oravka breed and supporting long-term conservation of one of the most valuable components of Slovakia’s national poultry genetic heritage (Table 2).

## 7. General Discussion

The conservation of native poultry breeds requires the integration of biodiversity preservation, reproductive biotechnology and sustainable breeding strategies [1]. Although considerable progress has been achieved in the cryopreservation of avian reproductive cells during the past two decades, no universal approach currently provides complete preservation of the avian genome [84]. The present literature review demonstrates that each biological material used for cryobanking possesses distinct advantages and limitations. BCs offer broad developmental potential but exhibit limited cryoresistance and germline transmission efficiency [77]. PGCs currently represent the most effective biological resource for preserving the complete genetic background of endangered breeds because they retain the capacity for germline transmission [35]. In contrast, MSCs constitute a valuable complementary resource for preserving somatic genomic information and supporting future applications in regenerative biology and functional genomics [50], whereas cryopreserved semen remains the most practical and routinely applicable material for gene bank implementation despite preserving only the paternal genome [64].

A major contribution of this review is the integration of these complementary cellular resources into a unified framework for conservation of the Slovak national chicken breed Oravka. Rather than discussing individual cryopreservation techniques independently, this review emphasises a multi-level conservation strategy in which different cell types collectively improve the long-term security of endangered poultry genetic resources. Furthermore, the manuscript incorporates recent findings from studies performed on the Oravka breed, providing practical evidence that complements the broader international literature and demonstrates how modern cryobiological methodologies can be successfully applied to a national genetic resource [84]. This integrated perspective may also serve as a model for the conservation of other local poultry breeds with limited population sizes.

The review also highlights several methodological challenges that remain insufficiently addressed. Differences in viability assessment methods, variability in cryoprotectant selection, the lack of standardised post-thaw quality evaluation, and breed-specific differences in cryotolerance continue to limit direct comparisons among published studies [70,84]. Greater standardisation of cryopreservation protocols and analytical methodologies will therefore be essential for improving reproducibility and facilitating the implementation of international poultry gene bank networks.

From a future perspective, rapid advances in reproductive biotechnology are expected to substantially enhance the efficiency of avian genetic conservation. Emerging technologies such as genome editing, single-cell transcriptomics, artificial intelligence-assisted optimisation of cryopreservation protocols, automated biobanking systems, and improved germline chimera production may significantly increase the success of breed restoration programmes [84]. In parallel, international collaboration among gene banks and harmonisation of conservation strategies will become increasingly important for safeguarding poultry biodiversity under climate change and emerging disease threats [1]. Integrating cryopreserved germline cells, somatic stem cells, semen, genomic information and comprehensive phenotypic databases into coordinated conservation programmes will provide a more resilient framework for preserving endangered poultry breeds for future breeding, research and sustainable agricultural production [1,84].

## 8. Conclusions

The conservation of native poultry breeds is essential for maintaining livestock biodiversity and strengthening the resilience of agricultural production systems in the face of climate change and changing breeding priorities. As one of Slovakia’s national genetic resources, the Oravka chicken represents an excellent model for demonstrating how traditional conservation programmes can be effectively complemented by modern reproductive biotechnologies, and it serves as a proof-of-concept for integrating national gene banking with advanced cryobiotechnology.

This review highlights that no single cryopreserved biological material is sufficient to ensure complete preservation of the avian genome. Instead, an integrated cryoconservation strategy combining PGCs, MSCs and cryopreserved rooster sperm provides the most comprehensive approach for safeguarding valuable genetic resources. While PGCs currently represent the most promising tool for preserving the complete genome through germline transmission, MSCs contribute additional opportunities for long-term biobanking and future biotechnological applications, and semen cryopreservation remains the most mature and readily applicable technology for routine gene bank programmes.

The integration of in situ and ex situ conservation strategies, supported by advances in cryobiology and stem cell technologies, will play an increasingly important role in protecting endangered poultry breeds. The Oravka chicken therefore represents not only an important component of Slovakia’s agricultural heritage but also a valuable model for developing integrated conservation frameworks applicable to other native poultry breeds worldwide. Continued research, methodological standardisation and international collaboration will be essential for ensuring that these genetic resources remain available for future breeding, research and sustainable food production.

## Figures and Tables

**Table 1:** Key publications on cryopreservation of poultry stem cells and germline cells.

Ref.	Biological Material	Main Contribution	Key Findings	Relevance for Oravka Conservation
[17]	Blastodermal cells	Description of EGK developmental stages	Established the developmental staging system used for isolation of BCs	Standard reference for embryonic cell collection
[18]	Blastodermal cells	Germline chimera production	Demonstrated limited germline transmission after in vitro culture	Shows limitations of BCs for gene banking
[19,20,21]	Blastodermal cells	Early cryopreservation studies	High post-thaw viability reported using Trypan Blue staining	Historical basis of BC cryobiology
[22]	Blastodermal cells	Flow cytometry and ultrastructure	Revealed lower true viability due to lipid-rich cytoplasm	Landmark study demonstrating limitations of BC cryopreservation
[23]	Blastodermal cells	Improved apoptosis assessment	Combined DRAQ5 with Annexin V to avoid false-positive staining	Improved methodology for viability evaluation
[25]	Primordial germ cells (PGCs)	Cryopreservation protocol	Successful slow freezing of gonadal PGCs with DMSO	Basis of modern poultry PGC banking
[26,27]	PGCs	Viability assessment	Demonstrated superiority of fluorescence staining over Trypan Blue	Standardised post-thaw quality assessment
[28]	PGCs	Cryobiology	Approximately 70% post-thaw survival with preserved ultrastructure and ALDH activity	Demonstrated excellent cryoresistance
[29,30,31,32,33,34]	Stem cells	ALDH marker	Established ALDH as a universal marker of stemness	Functional characterisation of cryopreserved cells
[35]	Oravka PGCs	Breed-specific PGC bank	Established stable male and female PGC lines with successful germline colonisation after cryopreservation	First comprehensive gene bank resource for the Oravka breed
[50]	Bone marrow MSCs	Oravka MSC cryobank	Isolation, characterisation and cryopreservation of Oravka MSCs	Complementary somatic genome preservation
[51,52,53,54,55,56,57,58,59,60]	Mesenchymal stem cells	MSC culture and cryopreservation	Confirmed high viability, multilineage differentiation and phenotype after freezing	Supports long-term MSC biobanking

**Table 2:** Landmark publications on poultry semen cryopreservation and animal genetic resource conservation.

Ref.	Topic	Main Contribution	Key Findings	Importance
[1]	FAO Animal Genetic Resources	Global conservation framework	International strategy for management of animal genetic resources	Fundamental reference for conservation policies
[2]	Cryopreservation	Overview of ex situ conservation	Summarised cryobanking approaches for animal genetic resources	General background
[63,64]	Rooster semen	Cryodamage after freezing	Demonstrated reductions in motility and fertilising capacity	Basis of poultry semen cryobiology
[65]	Cryoprotectants	Factors affecting semen freezing	Reviewed influence of CPA type, osmolarity and freezing protocols	Standard reference for protocol optimisation
[66]	Cryoprotectant toxicity	CPA-induced damage	Described cytotoxic effects of permeating cryoprotectants	Selection of optimal CPA
[67,68]	Oxidative stress	Mechanisms of cryoinjury	Identified oxidative stress as a major cause of sperm damage	Basis for antioxidant research
[69,70]	Breed differences	Cryotolerance	Demonstrated genotype-specific responses to freezing	Important for native breeds such as Oravka
[71,72,73,74]	CASA & Flow cytometry	Advanced sperm assessment	Introduced multiparametric evaluation of motility, viability, acrosome and mitochondrial function	Current gold standard for post-thaw evaluation
[75]	Glycerol cryopreservation	Comparative CPA study	Glycerol preserved highest sperm motility	Practical protocol optimisation
[77]	Oravka rooster semen	Multiparametric analysis	Compared DMSO, glycerol and ethylene glycol; introduced DRAQ5 for accurate viability assessment	Most relevant study for Oravka semen cryopreservation
[62]	Electron microscopy	Ultrastructural cryodamage	Identified plasma membrane as the primary target of freezing injury	Mechanistic understanding
[78,79]	Membrane biology	Lipid composition	Explained high cryosensitivity of avian sperm membranes	Biological basis of protocol development
[80,81,82,83,84,85]	Conservation biology	Native breeds and gene banks	Established integrated in situ and ex situ conservation strategies	Framework for national poultry gene banks

## Data Availability

The original contributions presented in this study are included in the article. Further inquiries can be directed to the corresponding author.
